# Exceptional performance of photoelectrochemical water oxidation of single-crystal rutile TiO_2_ nanorods dependent on the hole trapping of modified chloride

**DOI:** 10.1038/srep21430

**Published:** 2016-02-24

**Authors:** Xuliang Zhang, Haiqin Cui, Muhammad Humayun, Yang Qu, Naiying Fan, Xiaojun Sun, Liqiang Jing

**Affiliations:** 1 Key Laboratory of Functional Inorganic Materials Chemistry (Heilongjiang University), Ministry of Education, National Center for International Research of Catalytic technology, School of Chemistry and Materials Science, Harbin 150080, P.R. China; 2 Key Laboratory of Green Chemical Engineering and Technology of College of Heilongjiang Province, College of Chemical and Environmental Engineering, Harbin University of Science and Technology, Harbin 150040, P.R. China

## Abstract

It is highly desired to effectively trap photogenerated holes for efficient photoelectrochemical (PEC) water oxidation to evolve O_2_ on oxide semiconductors. Herein, it is found for the first time mainly based on the time-resolved- and atmosphere-controlled- surface photovoltage responses that the modified chloride would effectively trap photogenerated holes so as to prolong the charge lifetime and hence promote charge separation of single-crystal rutile TiO_2_ nanorods. Its strong capacity to trap holes, comparable to the widely-used methanol and Co(II) phosphate, is well responsible for the exceptional photoactivities for PEC water oxidation to evolve O_2_ on rutile nanorods with a proper amount of chloride modified, about 2.5-time high as that on the resulting anatase nanoparticles, even 10-time if the surface area is considered. Moreover, it is suggested that the hole trapping role of chemically-adsorbed chloride is related to its lonely-pair electrons, and to the subsequently-produced intermediate Cl atoms with proper electronegativity for evolving O_2_. Interestingly, this finding is also applicable to the chloride-modified anatase TiO_2_. This work will provide a feasible strategy to design high-activity nanostructured semiconductor photoanodes for PEC water oxidation, even for overall water splitting.

To realize the potential benefits of solar energy as a renewable energy source, it is desirable to develop a method to efficiently convert and store solar energy^1^,^2^,^3^. Since the first report of water splitting by a single-crystal rutile TiO_2_ photoanode from Fujishima and Honda over 40 years ago, the photocatalytic water splitting has been of particular interest as it can be used to produce H_2_ and O_2_^4^,^5^,^6^. Although the wide-bandgap TiO_2_ can only absorb ultraviolet (UV) light, its photocatalytic properties have been widely studied as a model system due to its low cost, high stability and thermodynamically-proper valence/conduction band energy levels for water reduction and oxidation to respectively evolve H_2_ and O_2_^1^,^7^,^8^. Among the three confirmed polymorphs of TiO_2_, anatase phase is the most popular one, usually with good photocatalytic performance, which is mainly attributed to its large surface area. On the contrary, rutile phase is seldom investigated owing to its small surface area and hence low photocatalytic activities^9^,^10^,^11^,^12^. This is because that the rutile phase is prepared via the traditional method of calcinating anatase crystallites with a high temperature (>550 °C), in which the rutile phases begin to occur at the interfaces of the aggregated anatase nanoparticles and hence make the formed rutile greatly grow up^10^,^12^,^13^. In fact, rutile phase possesses several advantages over anatase, such as high chemical stability, narrow band gap and high refractive index^12^. This implies that rutile TiO_2_ might have a great potential for utilizing solar energy if it has a large surface area, especially with single-crystal structure and rod-like morphology facilitating the charge transportation and separation.

In addition, the low quantum efficiency for photocatalytic water splitting still greatly limits the wide use of TiO_2_^14^,^15^. This is usually attributed to the slow half-reaction processes between photogenerated holes and water molecules within s timescale, compared to the recombination processes of photogenerated electron-hole pairs within ~μs timescale. The slow reaction for O_2_ evolution is also much slower than that for H_2_ production within ~μs timescale^15^,^16^. Naturally, it is widely accepted that the slow reaction process, with loss of 4e^−^ and 4H^+^ from two H_2_O molecules to evolve O_2_, is considered to be the rate-determining step for overall water splitting^15^,^17^. Thus, it is highly desired to develop a feasible strategy to effectively trap the photogenerated hole to prolong its lifetime, and/or to simultaneously catalyze the water oxidation. As for this, cobalt-based co-catalysts have been well investigated for efficient PEC water oxidation on TiO_2_^18^,^19^,^20^. In those works, it has been clearly demonstrated that the modified Co species could trap photogenerated holes effectively to produce high-valence Co ions, further inducing oxidation reactions with water molecules and subsequently returning to the original-valence ones. Very recently, a metal-free carbon nanodot-carbon nitride nanocomposite with the impressive performance for photocatalytic water splitting has been successfully fabricated, in which the modified carbon nanodots could play important roles as co-catalysts for evolving O_2_^21^. In our previous works^16^,^22^, it is clearly demonstrated that the formed surface negative fields after modification with phosphoric acids could prolong the lifetime of photogenerated charges of nano-sized TiO_2_ and BiVO_4_ by trapping photogenerated holes, leading to the enhanced photoactivities for PEC water oxidation. It is evident that for overall water splitting, it is very necessary to promote the trapping of photogenerated holes much efficiently. Therefore, it is much significant to develop feasible strategies to effectively trap photogenerated holes.

Since the photogenerated electrons and holes are respectively taken as negative and positive charge carriers, it is understandable that they would easily be trapped by the formed opposite electrostatic fields on the surfaces. Surprisingly, it is confirmed based on the transient absorption spectra that the surface carried positive charges have almost no effects on the charge carrier lifetime of nanocrystalline TiO_2_ in pure water and its photocurrent density in the NaClO_4_ electrolyte, indicating that it is not beneficial for water oxidation to trap the photogenerated electrons^16^. This is related to the point that the reactions between electrons and water molecules are faster compared to those between holes and water molecules. Similar to the formed surface negative field, it is expected thatthe surface anionic polarization by modifying with proper-electronegativity nonmetallic anions, like Cl^−^, would be favorable to trap photogenerated holes so as to prolong the charge carrier lifetime and hence to improve the activity of PEC water oxidation. In addition, it is worthy of noting that some biologists had found in the mid-20^th^ century that the chlorine species exhibited essential effects on the rate of oxygen evolution in the photosynthesis process^23^,^24^. Naturally inspired, it is much interesting to confirm those assumptions. To the best of our knowledge, it has not been reported until now. Obviously, it would be of great significance from the scientific and engineering point of views in the photochemical energy conversion field.

Based on the above analyses, we try to deeply reveal the effects of modified chloride on the photogenerated hole trapping, charge carrier lifetime, charge separation and photoactivities for PEC water oxidation to evolve O_2_ on single-crystal rutile TiO_2_ as a model system, mainly by means of time-resolved- and atmosphere-controlled- surface photovoltage responses, especially in N_2_ atmosphere. Interestingly, it is clearly demonstrated for the first time that the modified chloride would effectively trap the photogenerated holes so as to prolong the charge lifetime and hence to promote the charge separation of rutile TiO_2_ nanorods, leading to the exceptional photoactivities for PEC water oxidation to evolve O_2_. Moreover, it is suggested that the photogenerated hole trapping ability of chemically-adsorbed chloride is related to its lone-pair electrons and the subsequently-produced intermediate Cl atoms with proper electronegativity for evolving O_2_. This work will provide a feasible strategy to design high-activity nanostructured semiconductor photoanodes for PEC water oxidation to produce O_2_, even for photocatalytic overall water splitting.

## Results

**Figure 1:**
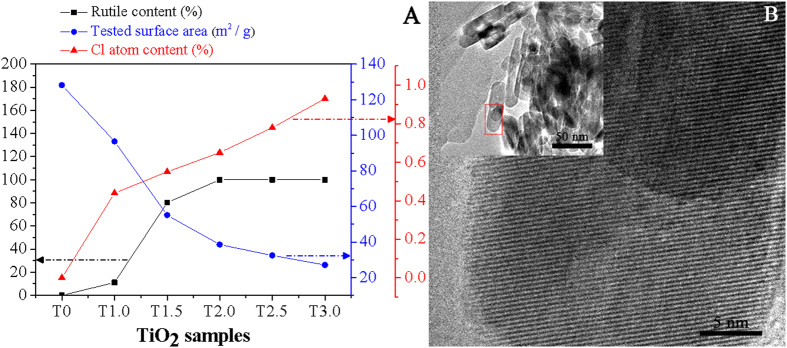
Rutile contents, tested BET surface areas, and residual Cl atom contents of different TiO_2_ samples prepared by varying the concentration of HCl solution used from 0 to 3.0 M (**A**), HRTEM image (**B**) and TEM image (**B** inset) of T2.5. TX indicates the resulting TiO_2_unless stated elsewhere, in which T means TiO_2_ and X is the concentration of HCl solution used.

We have prepared different anatase/rutile ratios of nanosized TiO_2_ by a HCl-added hydrothermal method^12^, in which the anatase/rutile ratios could be controlled by simply changing the concentration of HCl solution used as the structure-inducing agent by means of XRD patterns (Figure S1A) and related data (Fig. 1A). The XRD peaks at 2θ = 25.2° and 27.4° are often considered as the characteristic peaks of anatase (101) and rutile (110) phases, and the mass content percentage of rutile in two-phase-mixed TiO_2_ can be evaluated from the integrated characteristic peak intensities by means of the quality factor ratio^10^. It is noticed that the content percentage of rutile phase mainly depends on the concentration of HCl used, and it gradually become large with increasing the HCl amount. When the HCl concentration exceeds 2.0 M, the resultant product is only rutile phase TiO_2_. This is in good agreement with the UV-visible diffuse reflectance spectra (Figure S1B). Meanwhile, it is noticed that the specific surface area of the resulting TiO_2_ greatly decreases from 180 (pure anatase) to 30 m^2^/g (pure rutile). It should be pointed out that the obtained surface area of rutile is rather larger compared to that (generally <5 m^2^/g) of rutile prepared by traditionally calcining anatase nanoparticles. According to the TEM image (Fig. 1B inset), the obtained rutile nanoparticles display rod-like morphology, with ~20 nm in width and ~60 nm in length, and from the HRTEM (Fig. 1B), it is confirmed that the obtained rutile nanorod exhibits a single-crystal structure. Thus, it is naturally expected that the rutile phase composition, large surface area and nanorod structure of the as-prepared TiO_2_ are much beneficial for efficient photocatalytic reactions.

According to the XPS spectra of Ti2p, O1s, and Cl2p of different TiO_2_ (Figure S2), it is confirmed that, as the concentration of used HCl is increased, the binding energies (458.4 eV for Ti2p_3/2_ and 464.1 eV for Ti2p_1/2_) of Ti2p, resulting from Ti^4+^, gradually increased, whereas that of O1s is almost unchanged. The XPS spectra of O1s are broad and asymmetric, implying that there exists at least two kinds of oxygen chemical states, including crystal lattice oxygen (O_L_) centering at 529.5 eV and hydroxyl (O_H_) one centering at 531.5 eV^16^,^25^. The XPS peak at 197.8 eV is mainly assigned to the chemically-adsorbed chloride (Cl^−^) on the surfaces, and the amount of adsorbed chloride is increased as the rutile phase is gradually formed (Fig. 1A). Based on the chemical shift toward the higher binding energy of Ti2p, it is deduced that the chemically-adsorbed chloride on the surfaces is present in the form of –Ti-Cl by substituting surface hydroxyl groups. This well agrees with the point that the modified chloride by linking with Ti induces the direct formation of rutile in the synthesis of nanocrystalline TiO_2_^26^,^27^. Thus, the modified chloride would influence the surface physiochemical properties of rutile TiO_2_. Furthermore, from the EDX spectroscopy (Figure S1C), one can see that the chlorine exists in the T2.5 sample.

**Figure 2:**
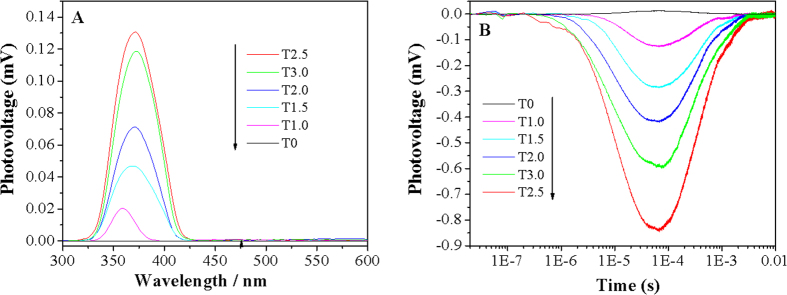
SS-SPS (**A**) and TR-SPV (**B**) responses of different TiO_2_ samples in N_2_ atmosphere.

To investigate the photogenerated charge properties of as-prepared TiO_2_, the steady-state surface photovoltage spectra (SS-SPS) and the time-resolved surface photovoltage (TR-SPV) responses are measured in N_2_ atmosphere, as shown in Fig. 2. It is well known that the SPS responses of nanosized semiconductor oxides mainly arise from the photoinduced charge carrier separation via the diffusion process^28^. Generally speaking, no SPS response for anatase or rutile TiO_2_ can be detected in N_2_ atmosphere since there is no any acceptor or donor species so that the surface charge amount before and after irradiation is unchanged. However, an obvious SPS signal can be detected in O_2_ atmosphere due to the adsorbed O_2_ as the acceptors to trap photogenerated electrons. Differently, the as-prepared TiO_2_ displays the gradually-enhanced SS-SPS responses in N_2_ with increasing the rutile phase content (Fig. 2A), and the complete rutile phase TiO_2_ (T2.5) exhibits the strongest SS-SPS response. Obviously, the observed SPS intensity mainly depends on the amount of modified chloride on the TiO_2_ surfaces. In the case, the adsorbed chloride would act as donators to trap the photogenerated holes so as to make corresponding electrons diffuse preferentially to the testing electrode surfaces^28^,^29^,^30^. However, if the residual-chloride amount is too much, it would be unfavorable for the surface charge transportation. As a result, the SS-SPS response tends to decrease, like T3.0 sample. More important, it is observed from Fig. 2B that all the rutile-containing TiO_2_ samples exhibit negative TR-SPV responses under laser pulse irradiation with wavelength 355 nm. This further confirms the role of modified chloride for trapping photogenerated holes. And also, the negative TR-SPV responseis gradually enhanced as the amount of modified Cl^−^ increased, with the prolonged charge lifetime, especially for the T2.5 sample. This is well consistent with the SS-SPS results.

**Figure 3:**
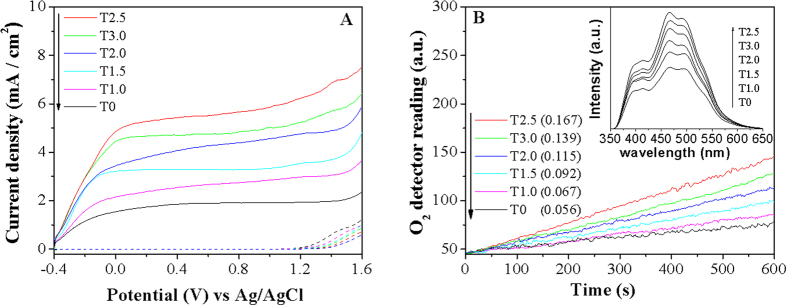
I–V curves (**A**) and produced O_2_ amount (**B**) of different TiO_2_ samples under illumination (solid-line) and in the dark (dash-line). Potentials are measured against an Ag/AgCl (saturated KCl solution) reference electrode in an oxygen-free 0.5 M NaClO_4_ solution. The number in the bracket indicates the rate constant of produced O_2_. Fluorescence spectra related to the formed hydroxyl radical amount after irradiation for 20 min is taken as the inset. The numbers in the brackets are the reaction constants for O_2_ evolution.

Based on the above analyses on the enhanced SPS/SPV responses, it is deduced that the modified chloride would effectively trap photogenerated holes so as to promote the separation of photogenerated charge carriers of TiO_2_. Hence, it is anticipated that the Cl-modified rutile nanorods would exhibit high photoactivities for PEC water oxidation. For this, we have also prepared the corresponding TiO_2_ films on the FTO glass substrates. Based on the XRD patterns (Figure S3A), it is confirmed that the phase composition of resulting TiO_2_ keeps unchanged. As expected, one can see from the SEM micrographs (Figure S3B) that the anatase TiO_2_ without Cl exhibits a spherical form with about 10 nm size, while the rutile TiO_2_ (T2.5) does a nanorod morphology, with the thickness of about 500 nm. As shown in Fig. 3A, it is noticed that no current is observed at the applied bias below 1.2 V vs Ag/AgCl standard electrode for all nano-sized TiO_2_ films. Interestingly, the photocurrent density is gradually increased as the rutile content (or the modified Cl amount) become large, and the rutile nanorod exhibits an exceptional photocurrent density. It is worthy of noting that the photocurrent density of T2.5 sample is enhanced by 2.5 times at 0.8 V bias compared to that of anatase one, accordingly being >10 folds if the surface area is considered. This is further supported by the amount of evolved O_2_ in the PEC water oxidation (Fig. 3B). Obviously, the photoactivity for PEC water oxidation to produce O_2_ is in good agreement with the SS-SPS (or TR-SPV) response intensity. In general, hydroxyl radical (·OH) is usually taken as the intermediate products for O_2_ evolution, and it is measured by the widely-used coumarin fluorescent method, in which the introduced coumarin easily reacts with ·OH to produce luminescent 7-hydroxycoumarin^31^. One can see from the inset in Fig. 3B that, as the amount of modified chloride is increased, the FS intensity related linearly to the amount of formed ·OH gradually become large, and that for T2.5 is the largest. However, over excess amount of modified Cl^−^ is unfavorable for further increase in the formed ·OH radicals, as for T3.0. This is well consistent with the evolved O_2_ amount.

## Discussion

**Figure 4:**
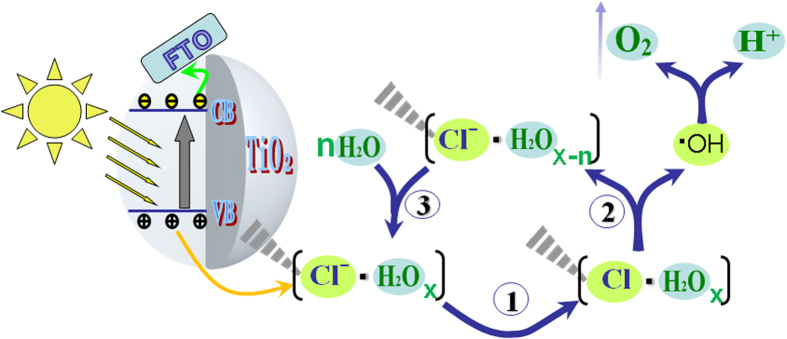
Schematic illustration of photogenerated holes transfer and its reaction with water molecule to evolve O_2_on the Cl-modified rutile TiO_2_. It is mainly involved with three steps. First step is that the modified chloridetraps photogenerated holes to form the Cl atoms [−Cl·H_2_O_x_] as fixed radicals. Second step is that the formed Cl atoms oxidize the complex water molecules to produce ·OH, subsequently followed by O_2_ evolution, meanwhile changing to the anionic state [−Cl^−^·H_2_O_x−n_]. The last one is that the anionic statereturns to its original form, [−Cl^−^·(H_2_O)_x_], by coordinating water molecules.

Based on the above results, it is naturally deduced that the modified chloride would play important roles in trapping photogenerated holes to promote charge separation and further initiating water oxidation to evolve O_2_. Thus, a possible mechanism schematic closely related to the modified-Cl role for PEC water oxidation is depicted in Fig. 4. As for this schematic, the modified chloride is firmly fixed on the surfaces in the form of [−Cl^−^·H_2_O_x_]^32^, so as to trap photogenerated holes to form the Cl atoms [−Cl·H_2_O_x_] as fixed radicals (step 1). Then, the formed Cl atoms oxidize the complex water molecules to produce ·OH (step 2), subsequently followed by O_2_ evolution, meanwhile changing to the anionic state [−Cl^−^·H_2_O_x−n_]. Finally, it would return to its original form, [−Cl^−^**·**(H_2_O)_x_] by coordinating water molecules (step 3). Differently from Cl modification, the improved photocatalytic performance of TiO_2_ by modification with F^−^, SO_4_^2−^ and PO_4_^3−^ mainly depends on the formed negative electric field, which could induce positive holes so as to promote photogenerated charge separation^16^,^22^,^33^. To well support the possible mechanism, the following experiments have been designed and completed.

One can notice from Figure S4 that the exceptional photocurrent density of resulting Cl-modified rutile nanorod keeps stable, and it is almost not influenced by the substitution electrolyte of NaClO_4_ solution and Na_2_SO_4_, both frequently employed as electrolytes^6^,^15^,^34^. This indicates that the observed large photocurrent is not correlated with the used electrolyte, and the modified chloride is fixed stably on the surfaces of TiO_2_. We have also synthesized the single-phase rutile TiO_2_ (called T0–800) by calcining the nanocrystalline anatase (T0) at 800 °C according to the XRD patterns(Figure S5A). As expected, no SS-SPS response for resulting T0-800 is observed in N_2_ atmosphere (Figure S5B), indicating that there are no donators to trap the photogenerated holes^35^. This leads to the rather small photocurrent density of T0-800 (Figure S5C), as compared to T0. Moreover, after post treating by a hydrothermal process at 160 °C for 2 h, the rutile crystallinity of T2.5 sample keeps almost unchanged, while the chemically-adsorbed chloride on the surfaces is mostly removed, denoted as T2.5-Cl-free, as shown in the XRD patterns (Figure S6A) and Cl2p XPS (Figure S6B). Differently from T2.5, the T2.5-Cl-free sample does not exhibit a detected SS-SPS response in N_2_ (Figure S6C). This is reasonably responsible for the greatly-decreased photocurrent density (Figure S6D). Obviously, it is clearly confirmed based on the above-designed experiments that the modified chloride is vital for greatly trapping photogenerated holes and hence for efficient water oxidation to evolve O_2_.

Thermodynamically, it is easily understandable that the photogenerated holes on TiO_2_ are trapped by the adsorbed chloride to accordingly form atomic Cl with proper electronegativity, which possesses enough capacity to oxidize water. In our previous work^36^, it was suggested that the borate groups could be modified on the surfaces of Cl-adsorbed rutile nanorods via the coordination bonds between lonely-pair-electron Cl and orbit-unoccupied B based on the XPS measurements, leading to the promotion of O_2_ adsorption and then photocatalytic activity for degrading colorless pollutants. Surprisingly, although the rutile phase keeps unchanged after borate modification (Figure S7A), the SPS attributeis changed (Figure S7B), to say that an obvious SPS response is disappeared in N_2_. Consequently, the photoactivity for PEC water oxidation is markedly decreased as shown in Figure S7C. Therefore, it is concluded that the lonely-pair electrons at the modified chloride is necessary conditions to trap photogenerated holes.

To further prove the above-mentioned roles of modified chloride, we have also carried out the chloride modification to the anatase TiO_2_ (T0), obtained as T0-XCl, by a hydrothermal process at 160 °C for 6 h in the presence of different concentration of NaCl solution. From the XRD patterns (Figure S8A) and UV-vis DRS spectra (Figure S8B), it can be seen that the chloride modification does not change the crystal phase and crystallinity of anatase TiO_2_. The XPS spectra (Figure S8C) indicate that the amount of modified chloride becomes large with increasing the NaCl concentration. As expected, the SS-SPS responses for different T0-Cl samples could be detected in N_2_ (Figure S8D), and its intensity is dependent on the amount of modified chloride. This is responsible for the enhanced photoactivity of anatase TiO_2_ for PEC water oxidation to evolve O_2_ after a proper amount of chloride modification (Figure S8E,F). Therefore, it is further demonstrated that the modified chloride exhibits great effects on hole trapping, charge separation and water oxidation.

Since that is the case about the role of modified chloride, it is expected that the presence of Cl^−^ would have positive effects on the photocurrent of anatase TiO_2_. As shown in Figure S9A, the photocurrent density in the NaCl-containing electrolyte is significantly increased, which is naturally attributed to the adsorbed Cl^−^. This is in agreement with Li’s and Iguchi’s works^37^, in which it was demonstrated that the activities for photocatalytic water splitting and CO_2_ reduction in the presence of Cl^−^ were enhanced by the formed intermediate product of HClO, while the roles for effectively trapping photogenerated holes by the adsorbed chloride were neglected. However, it should be pointed that no oxygen evolution is observed(Figure S9B). By comparison, it is suggested that the firmly-modified chloride on the surfaces of rutile nanorods is much favorable to form the intermediate ·OH, different from the formed intermediate HClO.

Based on the above discussion, it is believable that the modified chloride could effectively trap the photogenerated holes so as to promote the charge separation of rutile TiO_2_. To reflect its capacityto trap photogenerated holes, we have completed two confirmatory tests. Firstly, a certain concentration of methanol, often used as the hole scavenger, is introduced into the electrolyte during the PEC measurements of T2.5-Cl-free. As shown in Figure S10, the chloride-modified T2.5 exhibits the nearly same capacity to trap holes as the T2.5-Cl-free in the 0.01M methanol electrolyte. Secondly, we have taken the widely-employed cobalt (II) phosphate to modify T2.5-Cl-free as a ref. 18 Interestingly, it is confirmed according to Figure S11A, that the photocurrent of T2.5 is higher than that of the cobalt (II) phosphate-modified T2.5-Cl-free. Thus, it is evident that the capacity of modified chloride on rutile nanorods to trap photogenerated hole is rather high. For further comparison, we calculated the photon-to-current efficiency (IPCE) values of T0 and T2.5 using our PEC system under 365 nm wavelength illumination at bias of 0.8 V (vs Ag/AgCl) (Figure S11B). The result shows that the IPCE of T2.5 is 2.5-time larger than that of T0.

In summary, it is concluded that the modified chloride on the surfaces of rutile TiO_2_ nanorods could effectively trap photogenerated holes so as to promote the charge separation, leading to the exceptional photoactivities for PEC water oxidation to evolve O_2_. Its capacity to trap holes is comparable to the widely-employed methanol and Co(II) phosphate. The key of modified chloride is its lonely-pair electrons for trapping holes and its firm immobilization for keeping stable to form the intermediate Cl atoms and then ·OH groups for O_2_ evolution. This work would help to deeply understand the photophysical processes, and provide a feasible strategy to improve the photoactivities for efficient water oxidation on semiconductors.

## Methods

### Synthesis of materials

For the synthesis of rutile TiO_2_, the precursor (Tetrabutyl titanate) was added dropwise to a preferred concentration of HCl solution, kept in an ice bath, while maintaining the temperature below 10 °C. The reaction mixture was transferred to water bath and heated at 80 °Cfor 4 h so as to obtain white suspension. Subsequently, the suspension was transferred to stainless steel Teflon-lined autoclave and then followed by hydrothermal treatment at 160 °C for 6 h. After that, the resultant white precipitate was collected and washed with isopropanol followed by distilled water to remove the organic species and chloride ions. Finally, the product was dried at 100 °C for 12 h and referred as TX, in which X represents the molar concentration of HCl used and T represents the amount of TiO_2_^12^. For further exploration, first we calcined the T0 sample at 800 °C and obtained a kind of rutile TiO_2_. Then the T2.5 sample was hydrothermally treated in a stainless steel Teflon-lined autoclave at 160 °C for 6 h and washed repeatedly with isopropanol followed by distilled water, and finally dried at 100 °C for 12 h. In the final step, we added 0~1.0 g NaCl into 35 ml deionized water and stirred for 10 min, then added 0.5 g of T0 sample to the NaCl solution and stirred for 1 h. The mixture was transferred to the stainless steel Teflon-lined autoclave and hydrothermally treated at 160 °C for 6 h, then washed repeatedly with isopropanol followed by distilled water, finally dried at 100 °C for 12 h. The samples were referred as T0-YNaCl, in which Y indicates the amount of NaCl used in this process.

### Film preparation

To fabricate the films for photoelectrochemical (PEC) measurements, the corresponding pastes were synthesized as follows: 0.5 g nanopowder was dispersed in 2 mL iso-propyl alcohol, and then treated by an ultrasonic process for 30 min and stirring for 30 min. Then, 0.25 g of Macrogol-6000 was added to the above system, and retreated by an ultrasonic process for 30 min and stirring for 30 min. Subsequently, 0.1 mL of acetylacetone was introduced to the above mixture, followed by a 30 min-ultrasonic treatment and continuously stirring for 24 hours, forming the paste. Conductive fluorine doped tin oxide (FTO)-coated glasses, used as the substrates for the films, were cleaned by sonicating in acetone for 30 min and then in deionized water for another 30 min. After dried in air, the FTO was sintered at 450 °C for 30 min prior to use. Typically, the films were prepared by the doctor blade method using Scotch tape as the spacer, followed by drying in air for 30 min. Then, the films were sintered at 450 °C for 30 min. After that, the FTO glasses covered by the films were cut into 1.0 cm × 3.0 cm pieces with film surface area of 1.0 cm × 1.0 cm. To make a photoelectrode, an electrical contact was made with FTO substrate by using silver conducting paste connected to a copper wire which was then enclosed in a glass tube. The working geometric surface area was 0.5 cm × 0.5 cm where the remaining area was covered by epoxy resin^38^.

### Characterization of samples

The materials were characterized by using various techniques. The crystal structures of the samples were determined with the help of XRD (Bruker D8, Japan), using Cu Kα radiation (α = 0.15418 nm), at an accelerating voltage of 30 kV, and emission current of 20 mA was employed. The UV-vis diffuse reflectance spectra (UV-vis DRS) of the samples were recorded with Shimadzu UV-2550 Spectrophotometer, using BaSO_4_ as a reference. The surface composition and elemental chemical state of the samples were examined by X-ray photoelectron spectroscopy (XPS) using a Kratos-Axis Ultra DLD apparatus with an Al (mono) X-ray source, and the binding energies were calibrated with respect to the signal for adventitious carbon (binding energy 284.6 eV). The specific surface areas of the samples were measured by a Brunauer–Emmett–Teller (BET) instrument (Micromeritics Tristar II 3020 system) at the temperature of liquid nitrogen, while keeping the system out-gassed for 10 h at 150 °C prior to measurements. Scanning electron microscopy (SEM) micrographs were taken on a Hitachi S-4800 instrument operated at an accelerating voltage of 15 kV. Transmission electron microscopy (TEM) and energy dispersive X-ray spectroscopy (EDX) observations were carried out on a JEOL JEM-2010 instrument operating at a 300 kV accelerating voltage. The steady-state surface photovoltage spectroscopy (SS-SPS) measurement of the sample was carried out with a home-built apparatus that has been described elsewhere. Monochromatic light was obtained by passing light from a 500 W xenon lamp (CHFXQ500W, Global Xenon Lamp Power, made in China) through a double-prism monochromator (Hilger and Watts, D 300, made in England). A lock-in amplifier (SR830, made in USA), synchronized with a light chopper (SR540, made in USA), was employed to amplify photovoltage signal. The powder sample was sandwiched between two ITO glass electrodes, and the sandwiched electrodes were arranged in an atmosphere-controlled container with a quartz window for transmitting light.

The transient-state surface photovoltage (TS-SPV) response measurements were performed with a house-built device in N_2_ atmosphere at room temperature, in which the sample chamber is connected to an ITO glass as the top electrode and to a steel substrate as the bottom electrode, and an about 10 mm thick mica spacer was placed between the ITO glass and the sample to decrease the space charge region at the ITO-sample interface. The samples were excited by a 332 nm laser radiation with 10 ns pulse width from a second harmonic Nd:YAG laser (Lab-130-10H, Newport, Co.). The laser intensity was modulated with an optical neutral filter and measured by a high energy pyroelectric sensor (PE50BF-DIF-C, Ophir Photonics Group). The signals were registered by a 1 GHz digital phosphor oscilloscope (DPO 4104B, Tektronix) with an amplifier at the aid of a computer.

### Evaluation of activities

PEC experiment was performed in a glass cell with a quartz window using a 500 W xenon light with a stabilized current power supply as the illumination source and a 0.5 M NaClO_4_ solution as the electrolyte. The working electrode was the resulting TiO_2_ film (0.25 cm^2^ area), illuminated from the FTO glass side. A platinum plate (99.9%) was used as the counter electrode, and a saturated-KCl Ag/AgCl electrode (SSE) was used as the reference electrode. All the potentials in the paper were referred to the SSE. Oxygen-free nitrogen gas was used to bubble through the electrolyte before and during the experiment. Applied potentials were controlled by a commercial computer-controlled potentiostat (AUTOLAB PG STAT101).

To measure the amount of O_2_ evolved in the PEC water oxidation, the as-prepared films were used as working electrodes in a sealed quartz cell with 80 mL of 0.5 M NaClO_4_ solution as electrolyte, and high-purity nitrogen gas was bubbled through the electrolyte before the experiment. The films were illuminated from the FTO glass side, whose illuminated working area was about 1.5 × 1.5 cm^2^, at the constant bias of 0.8 V (vs Ag/AgCl). During the experiment, the amount of O_2_ evolved was detected quantitatively with an Ocean Optics fluorescence-based oxygen sensor (NFSC 0058) by putting the needle probe into the electrolyte near the working electrode, and the irradiation lasted for 10 min using a 500 W xenon light as the illumination source.

To measure the hydroxyl radical amount in the PEC condition, the as-prepared films were used as working electrodes in a sealed quartz cell with 80 mL of 0.5 M NaClO_4_/0.0002 M coumarin aqueous solution as electrolyte. The high-purity nitrogen gas was bubbled through the electrolyte before the experiment. The films were illuminated from the FTO glass side, whose illuminated working area was about 1.5 × 1.5 cm^2^, at the constant bias of 0.8 V (vs Ag/AgCl). After irradiation for 20 min, a certain amount of the solution was transferred into a Pyrex glass cell for the fluorescence measurement of 7-hydroxycoumarin at around 456 nm with 332 nm excitation through a spectrofluorometer (Perkin-Elmer LS55).

To measure the incident photon to current conversion efficiency (IPCE) in the PEC condition, the as-prepared films were used as working electrodes in a sealed quartz cell with 80 ml of NaClO_4_ solution as electrolyte. The high-purity nitrogen gas was bubbled through the electrolyte before the experiment. The films were illuminated from the FTO glass side, whose illuminated working area was about 1.5 × 1.5 cm^2^, at the constant bias of 0.8 V (vs Ag/AgCl). The monochromatic light was obtained by passing light from a 500 W Xenon lamp through a monochromator (CM110, Spectral Products).

## Additional Information

**How to cite this article**: Zhang, X. *et al*. Exceptional performance of photoelectrochemical water oxidation of single-crystal rutile TiO_2_ nanorods dependent on the hole trapping of modified chloride. *Sci. Rep.* **6**, 21430; doi: 10.1038/srep21430 (2016).

## Supplementary Material

Supplementary Information
