# Supplementary material for: Exceptional performance of photoelectrochemical water oxidation of single-crystal rutile TiO2 nanorods dependent on the hole trapping of modified chloride
Source: Sci Rep. 2016 Feb 24;6:21430. doi: 10.1038/srep21430 (PMC4764924; doi:10.1038/srep21430)

Electronic Supplementary Information (SI)

Exceptional performance of photoelectrochemical water oxidationof single-crystal rutile TiO_2 nanorods dependent on the hole trapping of modified chloride

Xuliang Zhang, Haiqin Cui, Muhammad Humayun, Yang Qu, Naiying Fan*, Xiaojun Sun*, and Liqiang Jing*

SI-Figures:

Figure S1 XRD patterns (A) and UV-vis spectra (B) of different TiO_2 samples prepared by varying the concentration of HCl solution used from 0 to 3.0 M. TX indicates the resulting TiO_2 unless stated elsewhere, in which T means TiO_2 and X is the concentration of used HClsolution. EDX spectroscopy (C).

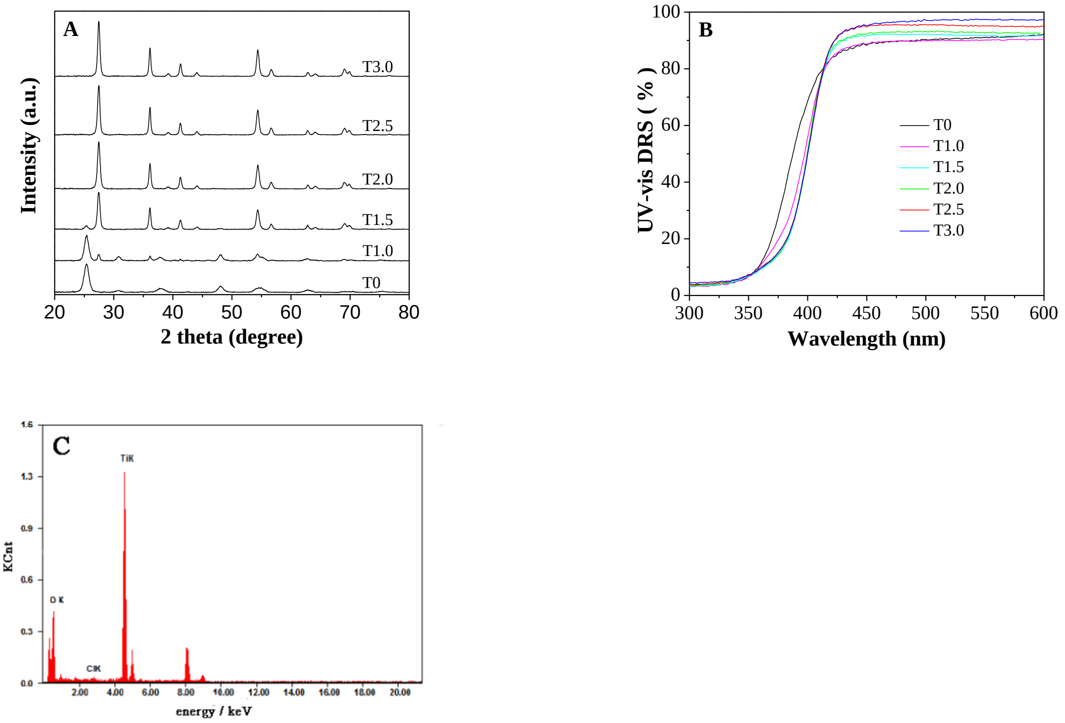

FigureS2 XPS spectra of Ti2p (A), O1s (B), and Cl2p(C) of different TiO_2samples.

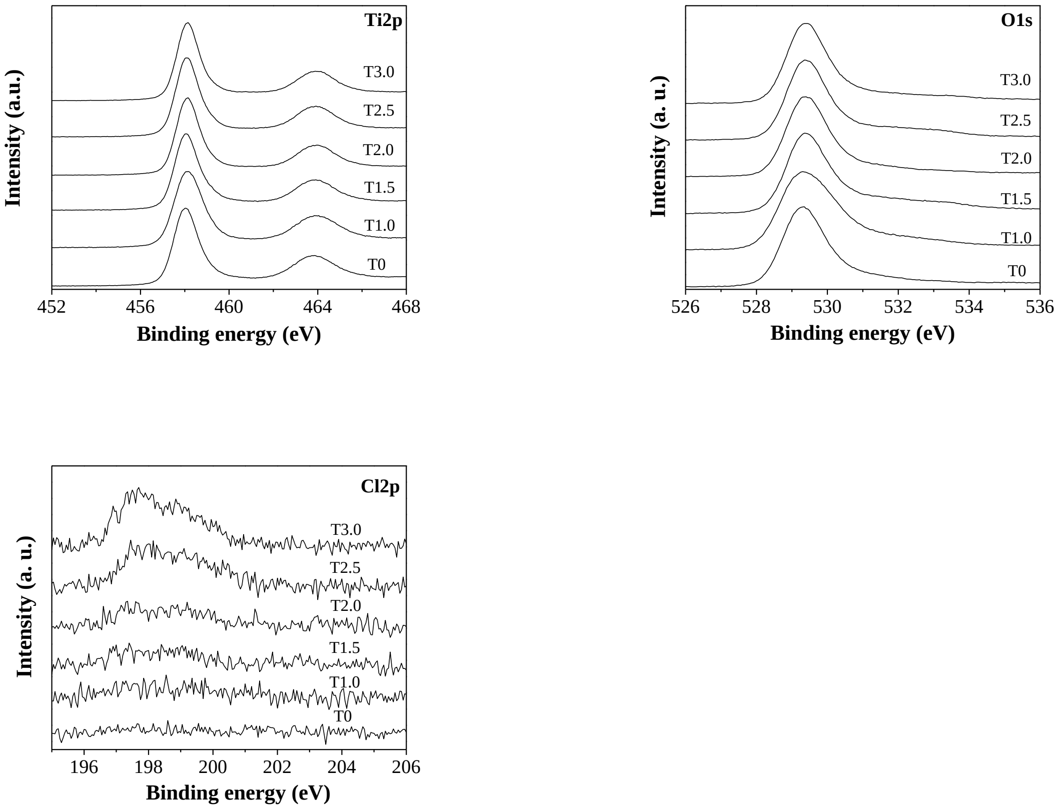

Figure S3 XRD patterns (A) and SEM images (B) of different TiO_2 films.

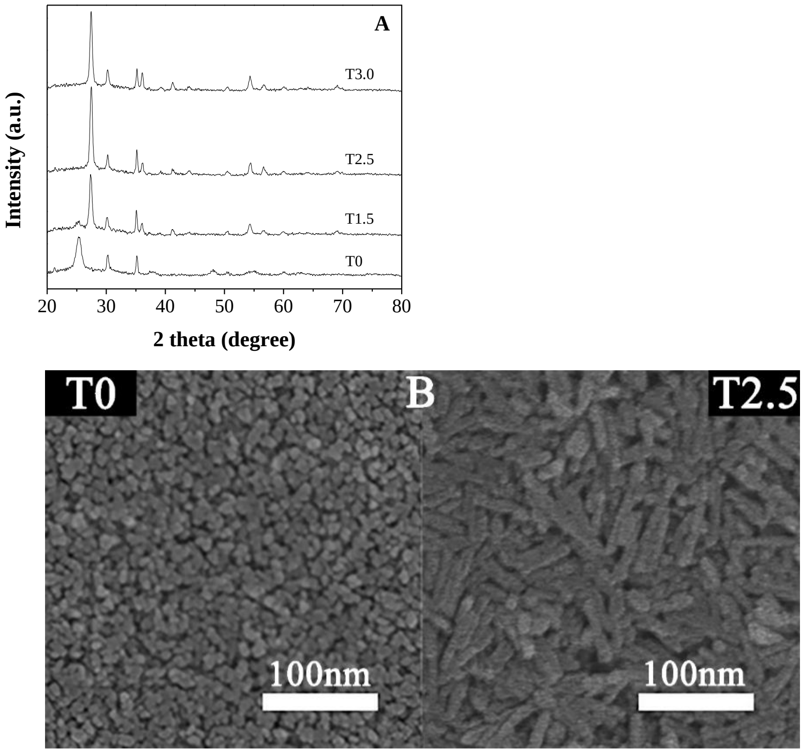

Figure S4 I-t curvesof T0 and T2.5 samples under illumination. Potentials are measured against an Ag/AgCl (saturated KCl solution) reference electrode in an oxygen-free 0.5 M $NaClO_4$(A) and Na_2SO_4(B) solution.

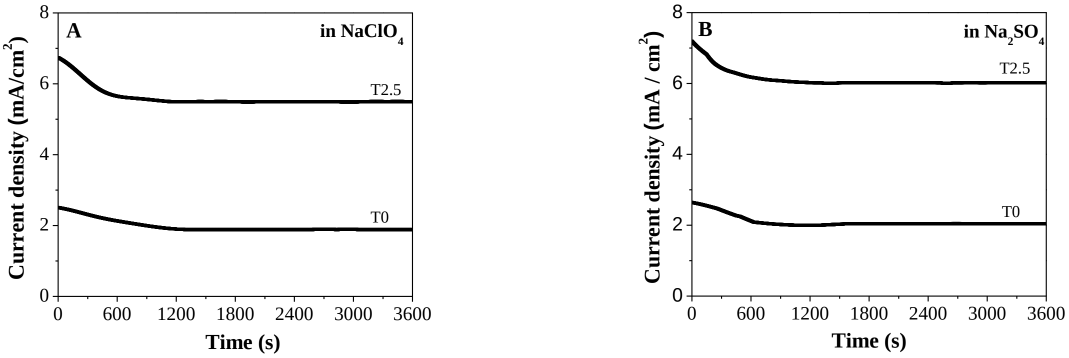

Figure S5 XRD patterns (A), SPS responses in different atmospheres (B), and I-V curves (C) ofrutile TiO_2 (T0-800) prepared by calcining T0 at 800 ℃. To compare, the T0 is also included here.

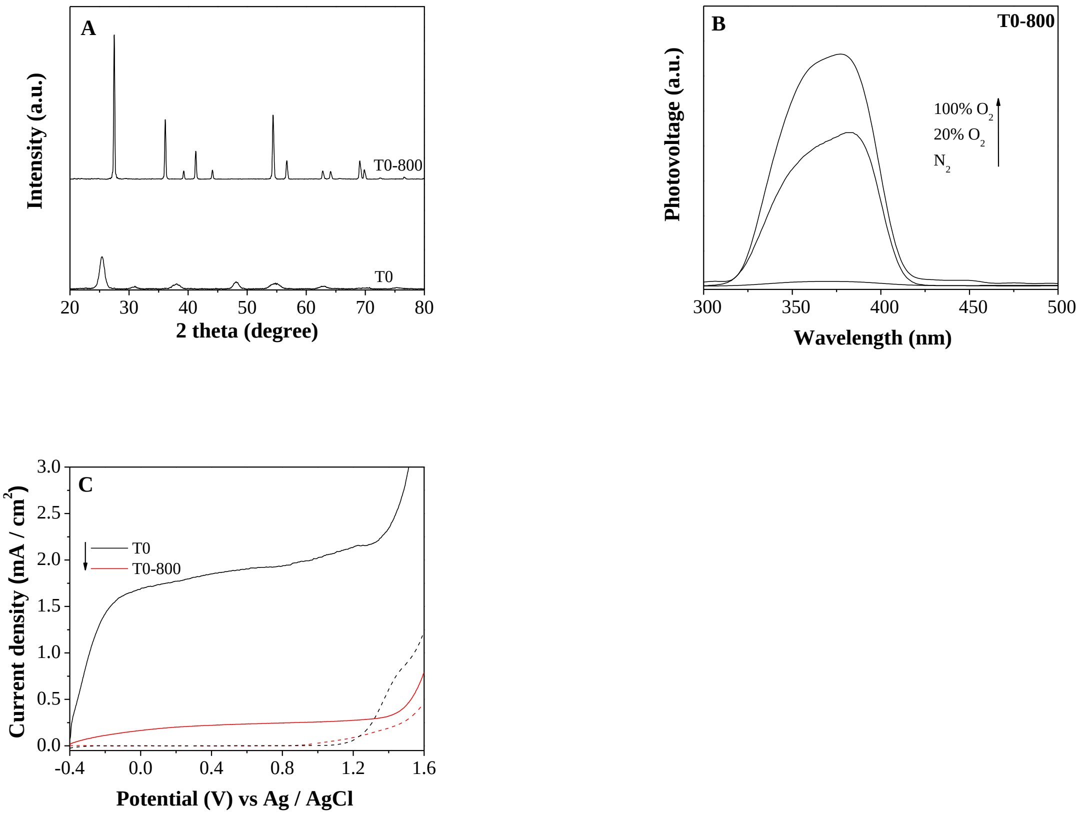

Figure S6 XRD patterns (A), XPS spectra of Cl2p (B), SPS responses in different atmospheres (C), and I-V curves (D) of TiO_2-Cl-free nanorod(T2.5-treated) treated by the twice hydrothermal process. To compare, the T2.5 is also included here. Potentials are measured against an Ag/AgCl (saturated KCl solution)reference electrodeinan oxygen-free 0.5 M $NaClO_4$solution. The SPS responses in different atmospheres of T2.5 is as taken as the inset.

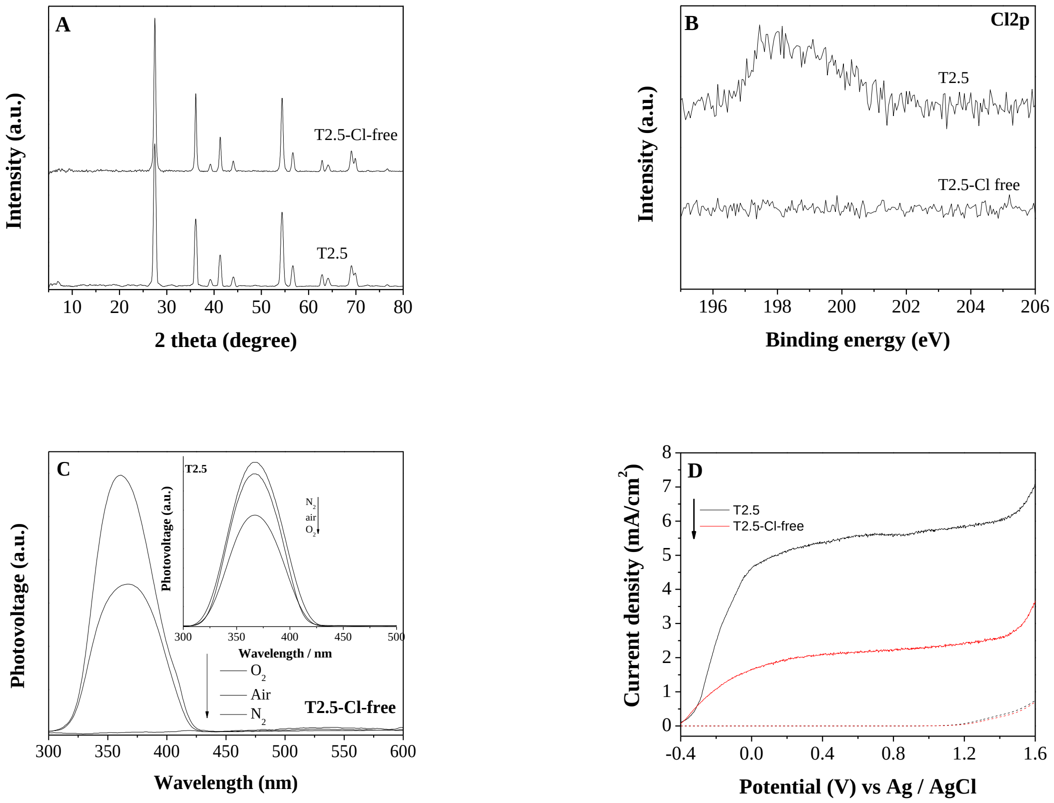

FigureS7 XRD patterns(A),SPS responses in different atmospheres (B),and I-Vcurves (C) of H_3BO_3-modifiedT2.5 (B-T2.5).Potentials are measured against an Ag/AgCl (saturated KCl solution)reference electrode in an oxygen-free0.5 M $NaClO_4$ solution.To compare, the T2.5 is also included here.

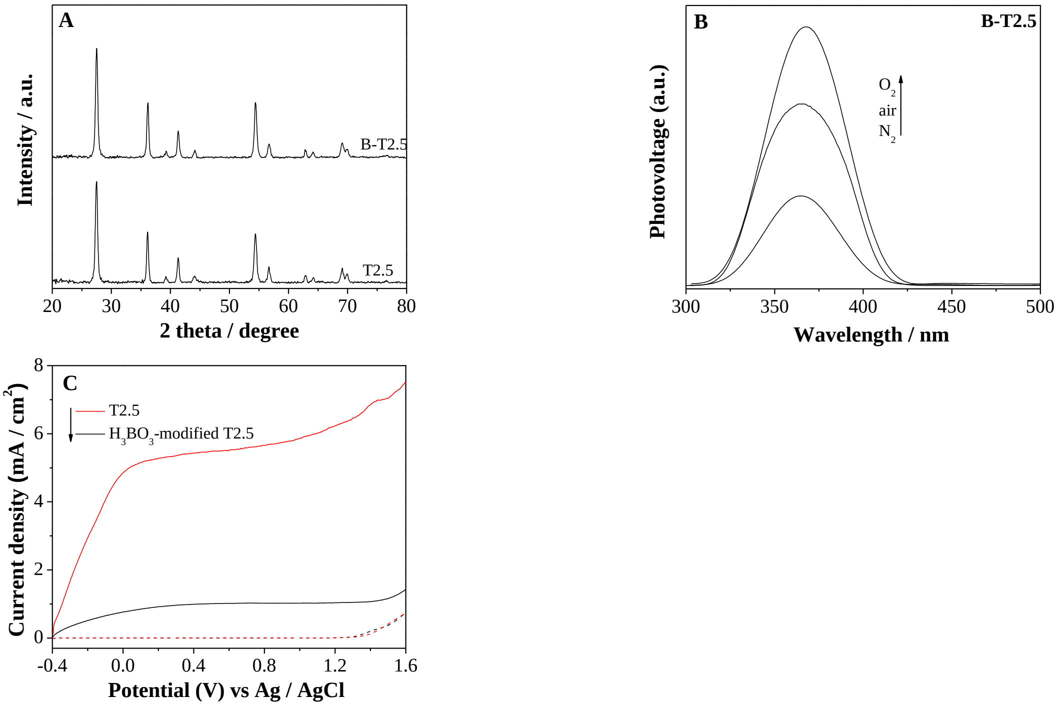

Figure S8 XRDpatterns (A), DRS spectra (B), Cl2p XPS spectra (C), SPS responses of T0-0.5NaCl in different atmosphere (D), I-V curves (E) and produced O_2 amount (F) of different chloride-functionalized anatase TiO_2 (T0-XNaCl), in which X indicates the mass of the NaCl added during the modification process. Potentials are measured against an Ag/AgCl (saturated KCl solution) reference electrode in an oxygen-free0.5 M $NaClO_4$solution.

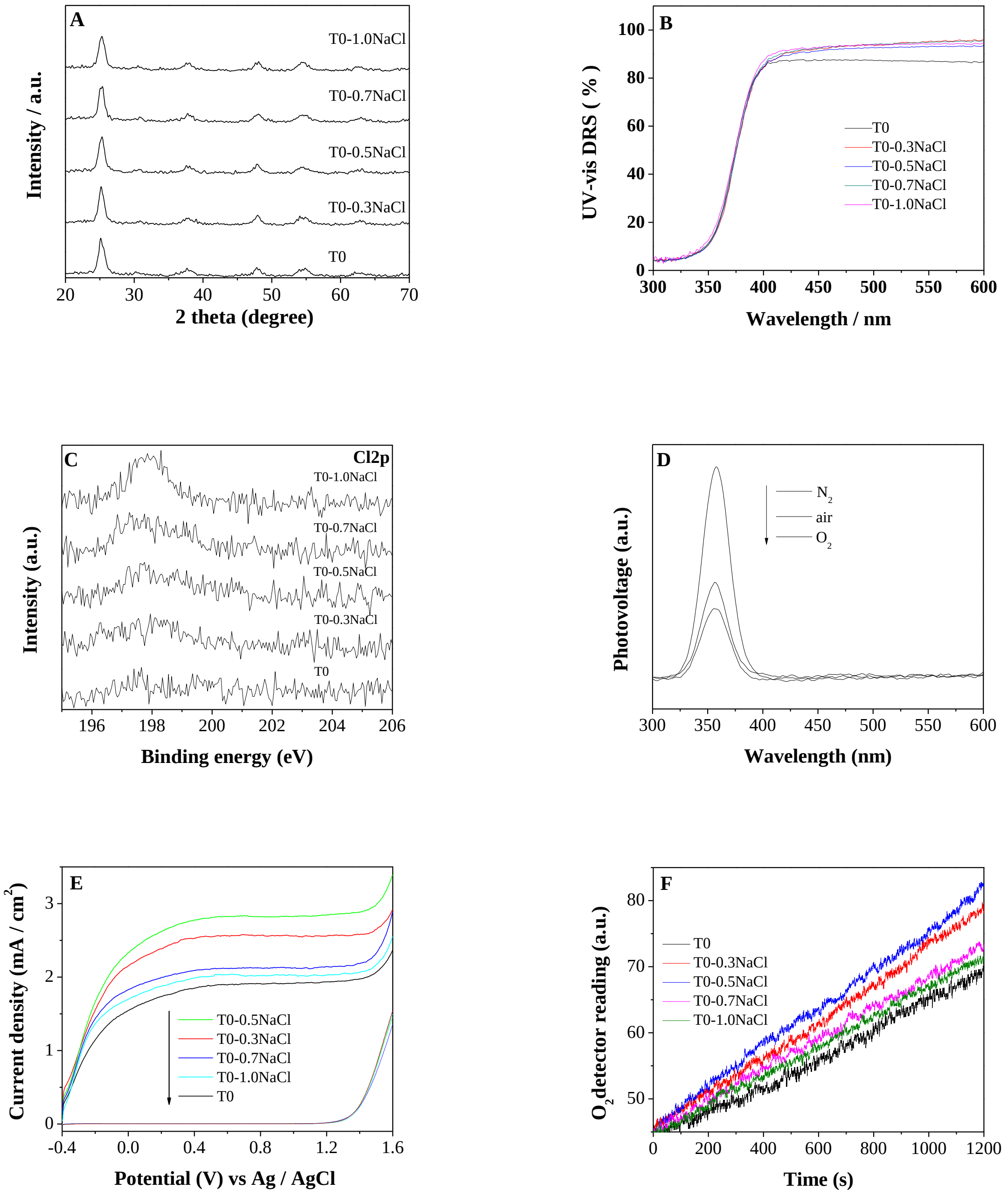

Figure S9 I-V curves (A) and O_2 evolution (B) of T2.5-Cl-free. Potentials are measured against an Ag/AgCl (saturated KCl solution)reference electrode in an oxygen-free0.5 M $NaClO_4$ solution and an oxygen-free 0.5 M $NaClO_4$ / 0.01 M NaCl solution.

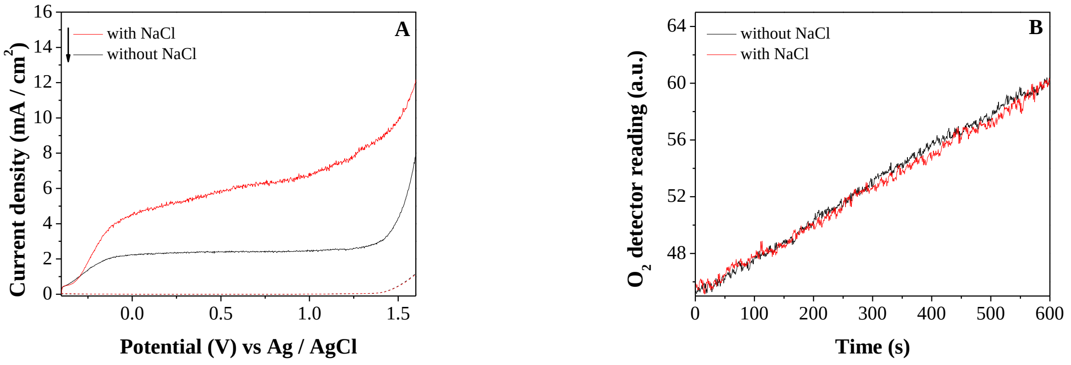

Figure S10 I-V curves under illumination (solid lines) and in the dark (dash lines). Potentials are measured against an Ag / AgCl (saturated KCl) reference electrode in a nitrogen purged 0.5M $NaClO_4$ (aq) solution. (a) T2.5, (b) T2.5-Cl-free, (c) 0.0045M Methanol-T2.5-Cl-free, (d) 0.009M Methanol-T2.5-Cl-free.

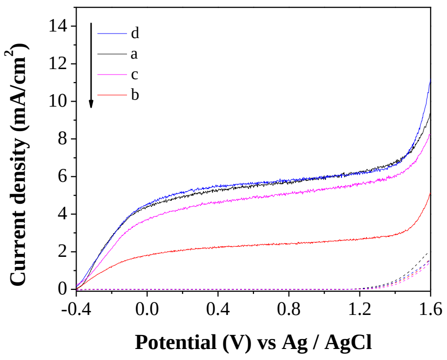

Figure S11 I-V curves under illumination (solid lines) and in the dark (dash lines) (A). Potentials are measured against an Ag / AgCl (saturated KCl) reference electrode in a nitrogen purged 0.5M $NaClO_4$ (aq) solution,(a) T2.5, (b) T2.5-Cl-free, (c) 2×10^{-3} Co-P-T2.5-Cl-free, (d) 5×10^{-3} Co-P-T2.5-Cl-free, (e) 8×10^{-3} Co-P-T2.5-Cl-free. IPCE value of T0 and T2.5 under different wavelength illumination (B).

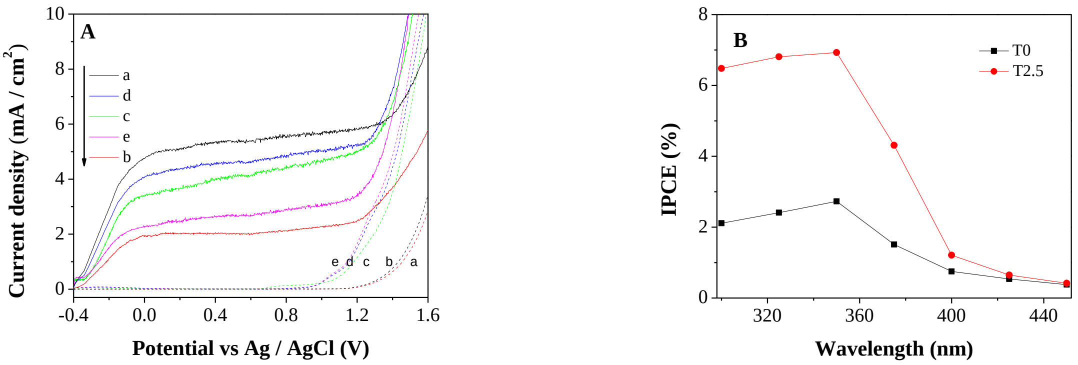

Supplement: Supplementary Information [file srep21430-s1.pdf]
